# Is radioiodine necessary for patients with low-risk differentiated thyroid cancer after thyroidectomy: a pooled analysis of ESTIMABL2 and IoN trials

**DOI:** 10.3389/fonc.2025.1670978

**Published:** 2025-10-28

**Authors:** Chuansheng Yang, Deping Luo, Jian Xie, Yingting Zou, Fei Chen, Wenchun Yang, Linfeng Zeng, Jing Liu

**Affiliations:** ^1^ Department of Nuclear Medicine, Ganzhou Cancer Hospital, Ganzhou, China; ^2^ Department of Gynecological Oncology, Ganzhou Cancer Hospital, Ganzhou, China; ^3^ Department of Medical Technology, Gannan Healthcare Vocational College, Ganzhou, China

**Keywords:** radioiodine, low-risk differentiated thyroid cancer, thyroidectomy, meta-analysis, randomized controlled trials

## Abstract

**Background:**

The clinical utility of postoperative radioiodine therapy in patients with low-risk differentiated thyroid cancer (DTC) remains a subject of ongoing debate. Although radioiodine has been widely employed to reduce the risk of recurrence, its necessity in low-risk populations is increasingly questioned, given the favorable outcomes observed with surgery alone. To address this issue, we conducted a meta-analysis exclusively based on randomized controlled trials (RCTs) to comprehensively evaluate the efficacy and safety of radioiodine therapy in this specific patient population.

**Methods:**

We systematically searched 6 databases for eligible phase 3 RCTs comparing surgery with or without radioiodine in patients with low-risk DTC. Primary outcomes included recurrence and recurrence-free survival (RFS); secondary outcomes included adverse events (AEs), structural events, and biological events. Risk ratios (RRs) or hazard ratios (HRs) with 95% confidence intervals (CIs) were pooled and analyzed.

**Results:**

Two phase 3 RCTs (the ESTIMABL2 and IoN trials), encompassing 1280 patients, were included. Compared to the non-radioiodine group, radioiodine therapy did not significantly reduce recurrence rates (RR: 0.78 [0.36-1.70], *P* = 0.53) or improve RFS (HR: 0.96 [0.80-1.15], *P* = 0.68). The total number of structural events (RR: 0.83 [0.68-1.02], *P* = 0.07) and biological events (RR: 0.88 [0.71-1.08], *P* = 0.23) were also similar between the two groups. In the safety analysis, the two groups exhibited comparable rates of AEs (RR: 0.97 [0.79-1.20], *P* = 0.80), grade 3–5 AEs (RR: 0.25 [0.03-2.20], *P* = 0.21), death (RR: 1.28 [0.48-3.41], *P* = 0.62), and second primary cancers (RR: 1.26 [0.58-2.73], *P* = 0.55).

**Conclusion:**

Radioiodine therapy did not confer significant benefits in reducing recurrence or improving RFS in patients with low-risk DTC after thyroidectomy, and the safety profiles were comparable between the two groups.

**Systematic Review Registration:**

https://www.crd.york.ac.uk/prospero/, identifier CRD420251105509.

## Introduction

Differentiated thyroid cancer (DTC) is the most prevalent endocrine malignancy, with papillary thyroid carcinoma accounting for approximately 85-90% of all cases (1). Over the past several decades, the global incidence of DTC has markedly increased, largely due to the widespread use of high-resolution imaging and enhanced diagnostic practices (2). Despite this rising incidence, the majority of patients—especially those with low-risk DTC—have an excellent prognosis, with disease-specific survival rates exceeding 95% at 10 years (3).

For low-risk DTC, which is typically defined by small, intrathyroidal tumors without lymph node involvement or distant metastasis, thyroidectomy alone is often considered curative. However, the postoperative use of radioiodine therapy in this population remains a matter of ongoing debate (4). Historically, radioiodine was widely administered to ablate remnant thyroid tissue, facilitate follow-up with serum thyroglobulin, and potentially eliminate microscopic residual disease (5). Yet, with the evolution of risk stratification systems and improved surveillance methods, the necessity of radioiodine in low-risk patients has been increasingly questioned (6).

Several studies and retrospective analyses have suggested that radioiodine provides little to no benefit in reducing recurrence or improving survival in low-risk DTC (7, 8). Moreover, radioiodine is not without risks. Acute and chronic adverse effects such as sialadenitis, taste alterations, and, in rare cases, second primary malignancies have raised concerns about overtreatment (9). These potential harms, combined with the excellent baseline prognosis of low-risk patients, have led to more conservative guideline recommendations. Notably, the 2015 American Thyroid Association (ATA) guidelines advise against the routine use of radioiodine in patients with low-risk DTC (10). To provide high-level evidence, two recent phase 3 randomized controlled trials—ESTIMABL2 and IoN—were conducted specifically in low-risk DTC populations to assess whether radioiodine therapy offers any additional clinical benefit (11, 12). These trials used modern diagnostic tools and long-term follow-up protocols, enabling a more accurate evaluation of recurrence and adverse outcomes.

In light of this, we performed a meta-analysis of available phase 3 RCTs comparing radioiodine therapy versus non-radioiodine in patients with low-risk DTC following thyroidectomy. Our objective was to clarify the impact of radioiodine on recurrence, recurrence-free survival (RFS), adverse events (AEs), mortality, and secondary cancer risk, and to provide comprehensive evidence to guide individualized treatment decisions for this growing patient population.

## Materials and methods

### Search strategy

A systematic search approach was applied using terms including “Radioiodine”, “Thyroid Cancer”, and “Randomized” (**Supplementary Table S1**), and relevant trials were retrieved from major databases such as PubMed, EMBASE, ScienceDirect, Cochrane Library, Web of Science, and Scopus, covering studies published until June 20, 2025.

### Selection criteria

The inclusion standards were:

(1) Patients confirmed with low-risk DTC (defined according to the WHO Classification of Endocrine and Neuroendocrine Tumours, 5th Edition [2022], Thyroid Tumours chapter: Non-invasive follicular thyroid neoplasms with papillary-like nuclear features, tumors of uncertain malignant potential, and hyalinizing trabecular tumors — all originating from follicular epithelial cells — are characterized by encapsulation or well-circumscribed borders and the absence of lymph node or distant metastasis [EX0, N0, M0]) (13);

(2) Comparisons of radioiodine and non-radioiodine after thyroidectomy;

(3) Documentation of at least one outcome: recurrence, RFS, AEs, structural events, and biological events.

(4) Phase 3 RCTs published in English;

Studies were excluded if they met any of the following criteria: (1) review articles, meta-analyses, or case-based descriptions; (2) experiments conducted on animals or non-human models; (3) data unavailable or insufficient for analysis.

### Data extraction

Two reviewers independently used a standardized data sheet to extract trial-related details. Information included study profiles (patients, histology, etc), recurrence, RFS, AEs, mortality, structural events, and biological events. Structural events in both cohorts referred to suspicious imaging results on neck ultrasound, such as abnormal nodes or thyroid remnants. Biological events were defined as elevated Tg or TgAb levels without structural confirmation. Any conflicting interpretations were discussed, and unresolved differences were adjudicated by a third investigator.

### Quality assessment

Assessment data were extracted from peer-reviewed publications and, when available, official trial registries. Methodological quality and risk of bias were evaluated using the Cochrane risk-of-bias tool and the Jadad scale (14, 15). Studies with Jadad scores ranging from 4 to 7 were considered to have high methodological quality. Additionally, the certainty of evidence was assessed using the GRADE framework, with evidence categorized from high to very low certainty (16).

### Statistical analysis

Statistical evaluations were conducted using RevMan 5.4 and STATA 17.0. Pooled estimates of hazard ratio (HR) and risk ratio (RR) were derived to examine event-time and dichotomous outcomes. Between-study variability was measured using the I² statistic and Cochran’s Q test, with heterogeneity deemed considerable when I² exceeded 50% or the p-value was below 0.10. A random-effects model addressed substantial heterogeneity, whereas fixed-effects were chosen for lower inconsistency. Funnel plots were utilized to assess reporting bias. Statistical significance was established at p < 0.05.

## Results

### Search results

**Figure 1:**
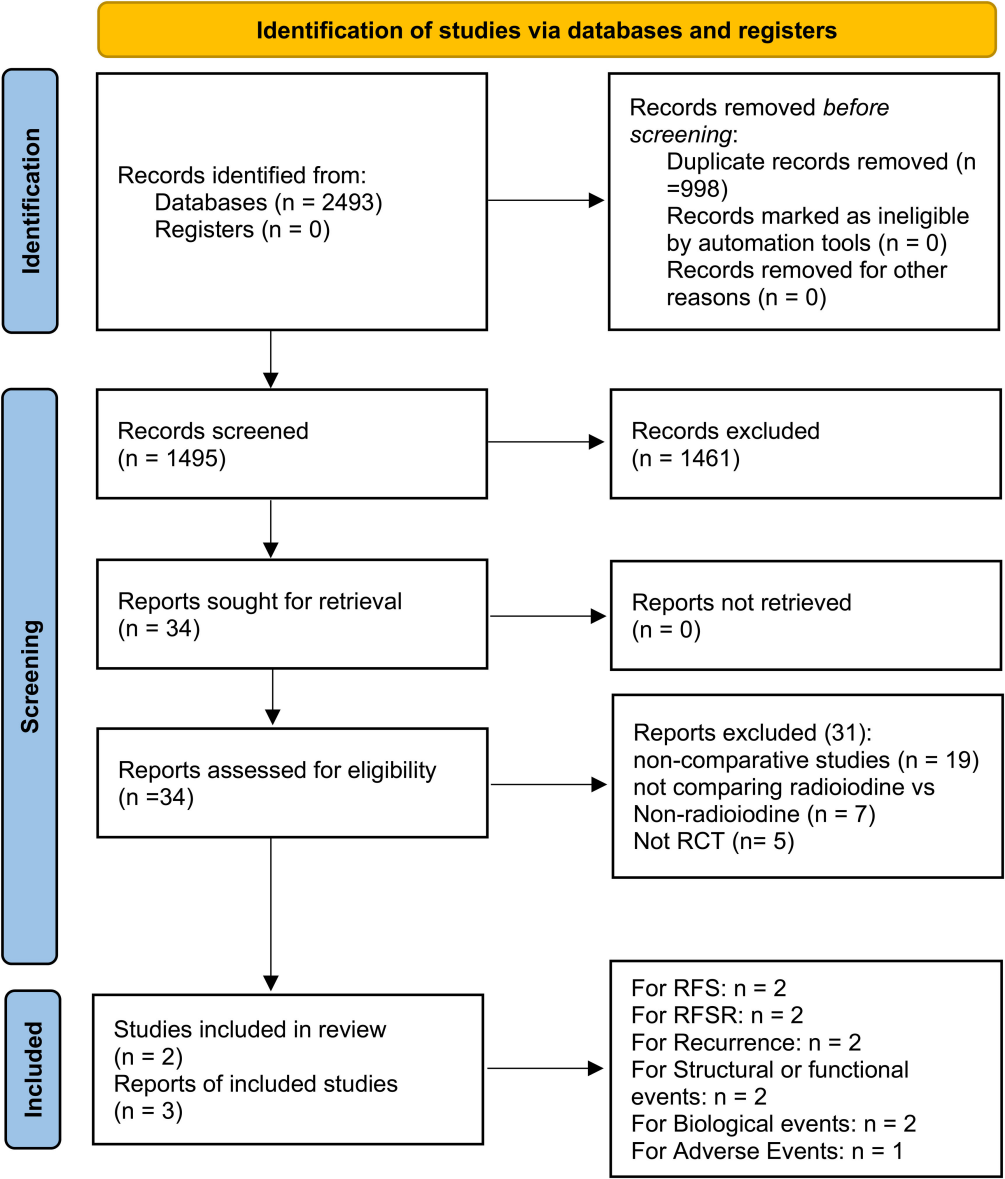
Flow chart.

This pooled review incorporated three reports derived from two phase 3 RCTs—ESTIMABL2 and IoN—encompassing 1280 individuals diagnosed with low-risk DTC (11, 12, 17). Study selection steps, detailed in **Figure 1**, adhered to PRISMA 2020 criteria. **Supplementary Figure S1** and **Supplementary Table S2** reveal both trials employed rigorous methodology and exhibited minimal bias. According to the GRADE framework, the certainty of evidence ranged from moderate to high (**Supplementary Table S3**). The ESTIMABL2 study was performed in France (11), while the IoN investigation took place in the United Kingdom (12). **Table 1** presents core trial structures and baseline participant characteristics.

**Table 1:** Baseline characteristics of the included studies.

Characteristics	ESTIMABL2		IoN	
Register number	NCT01837745		NCT01398085	
Design	RCT		RCT	
Clinical trial stage	Phase 3		Phase 3	
Inculded articles	Leboulleux 2025 (11), Leboulleux 2022 (17)		Mallick 2025 (12)	
Country	French		United Kingdom	
Period	2013.05-2017.03		2012.06-2020.03	
Treatment arms	Radioiodine	Non-radioiodine	Radioiodine	Non-radioiodine
Radioactive iodine activity	1.1 GBq	–	1.1 GBq	–
Patients (n)	389	387	253	251
Sex (M/F)	70/319	64/323	54/199	60/191
Median age (year)	52.2	52.9	47	48
Histology				
Papillary	372	372	204	192
Follicular	13	11	38	52
Oncocytic	4	4	11	7
Multifocality	78	156	97	89
Stage				
pT1	389	387	118	117
pT2	0	0	112	111
pT3	0	0	23	23
Nodal status				
Nx	220	216	57	57
N0	169	171	172	171
N1a	0	0	24	23
Central compartment neck dissection	143	142	40	51
Follow-up duration (months)	60	60	79.2	81.6

M/F, Male/Female; RCT, Randomized controlled trial.

### Recurrence

**Figure 2:**
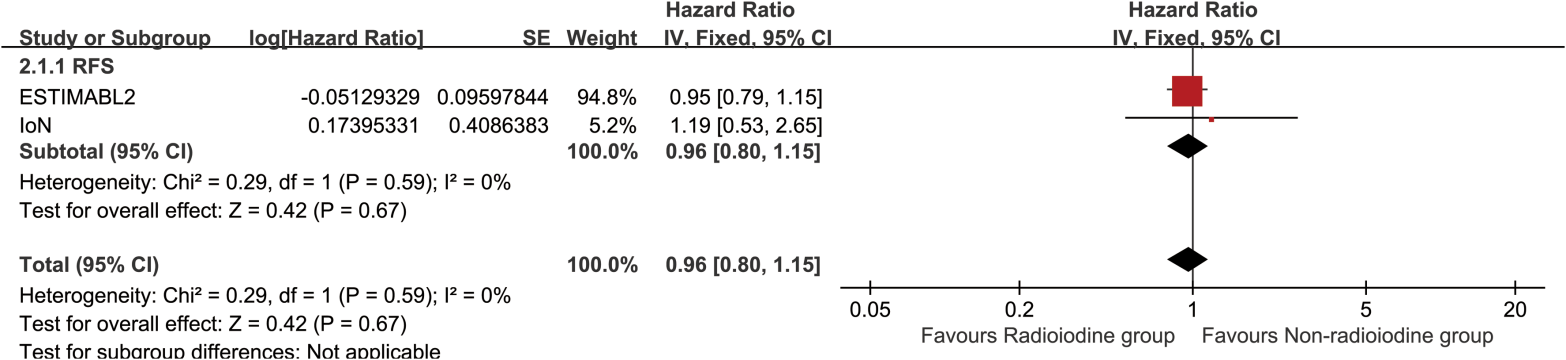
Forest plot of recurrence-free survival associated with radioiodine versus non-radioiodine.

**Figure 3:**
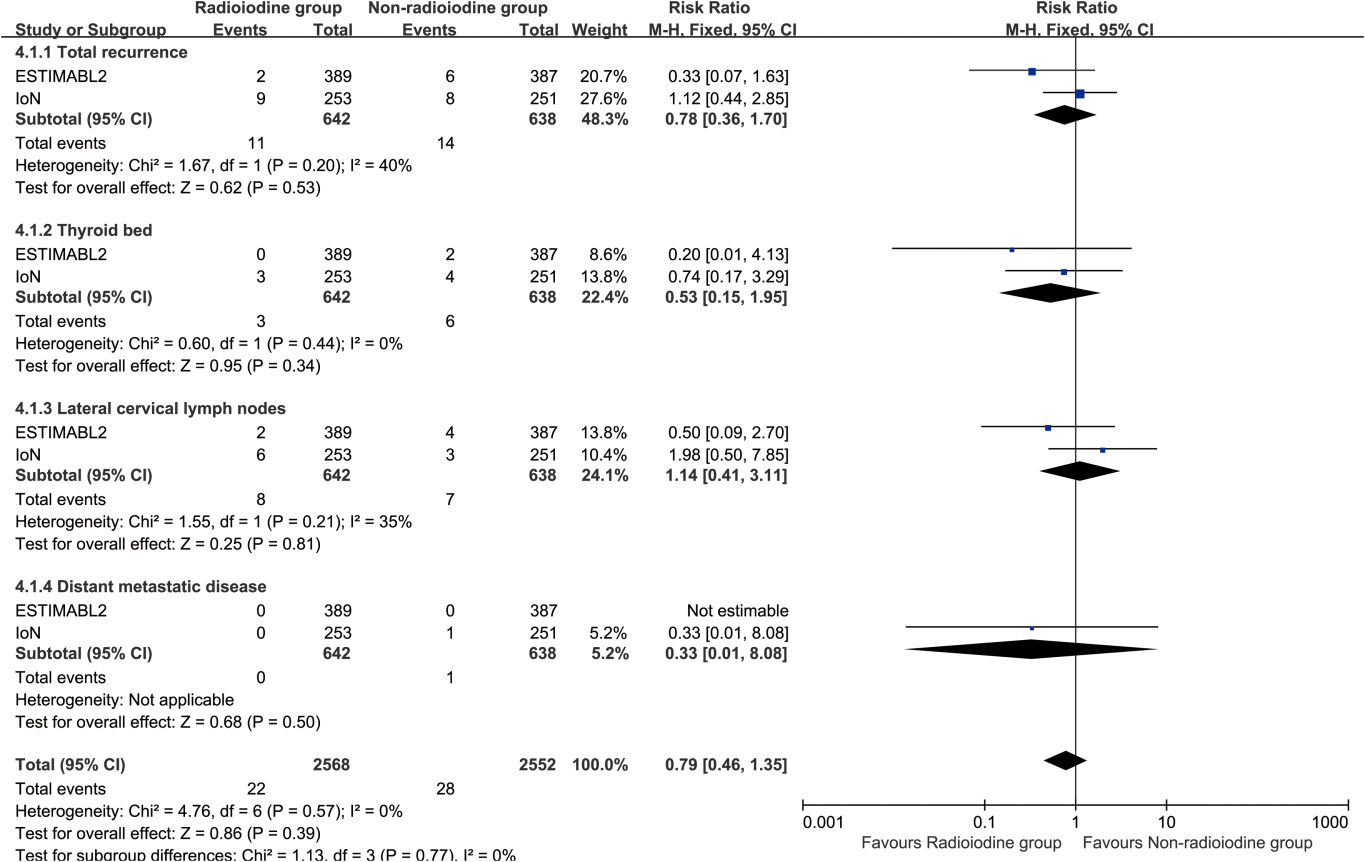
Forest plot of recurrence associated with radioiodine versus non-radioiodine.

Radioiodine therapy did not significantly improve RFS (HR: 0.96 [0.80–1.15], P = 0.68) (**Figure 2**). Between 1 and 5 years, the RFS rates (RFSR) were similar in both groups (**Supplementary Figures S2**, **S3**). Likewise, the total recurrence rate and site-specific recurrence rates did not differ significantly between the two groups (**Figure 3**).

### Structural and biological events

**Figure 4:**
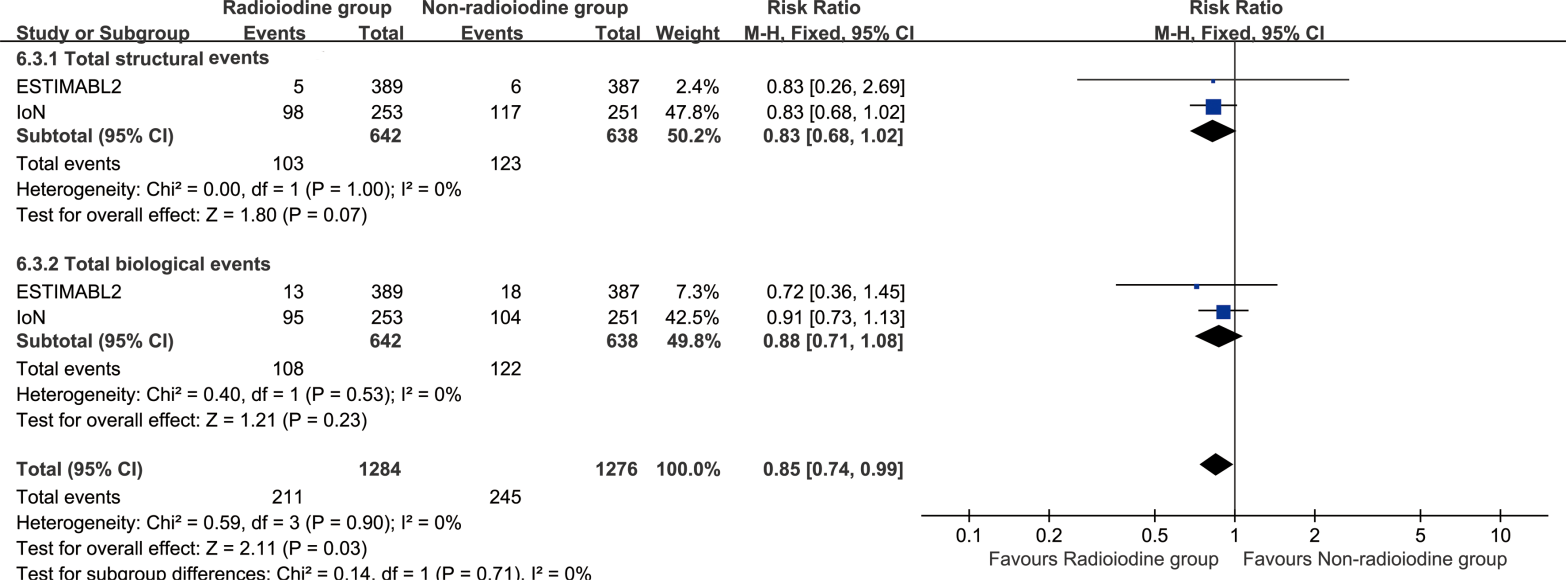
Forest plot of total structural events and biological events associated with radioiodine versus non-radioiodine.

Total structural events (RR: 0.83 [0.68, 1.02], P = 0.07), and biological events (RR: 0.88 [0.71, 1.08], P = 0.23) were comparable between the two groups (**Figure 4**). Notably, Tg > 5ng/ml at any time point was more frequently observed in the non-radioiodine group (**Supplementary Figure S4**). Subgroup analyses of structural and biological events also revealed no significant differences between the two groups (**Supplementary Figure S5**).

### Adverse events

Both any grade AEs (RR: 0.97 [0.79, 1.20], P = 0.80) and grade 3–5 AEs (RR: 0.25 [0.03, 2.20], P = 0.21) were comparable between the two groups. No statistically significant differences were observed for any individual AE (**Table 2**, **Supplementary Table S4**). The three most frequently reported AEs in the radioiodine group were fatigue (25.69%), lethargy (12.65%), and dry mouth (8.30%).

**Table 2:** Any grade adverse events.

Adverse events	Radioiodine		Non-radioiodine		Risk ratio [95% CI]	P
	Event/total	%	Event/total	%		
Total	103/253	40.71%	105/251	41.83%	0.97 [0.79, 1.20]	0.80
Fatigue	65/253	25.69%	63/251	25.10%	1.02 [0.76, 1.38]	0.88
Lethargy	32/253	12.65%	34/251	13.55%	0.93 [0.60, 1.46]	0.77
Dry mouth	21/253	8.30%	24/251	9.56%	0.87 [0.50, 1.52]	0.62
Depression	18/253	7.11%	16/251	6.37%	1.12 [0.58, 2.14]	0.74
Dizziness	16/253	6.32%	12/251	4.78%	1.32 [0.64, 2.74]	0.45
Headache	14/253	5.53%	17/251	6.77%	0.82 [0.41, 1.62]	0.56
Nausea	13/253	5.14%	8/251	3.19%	1.61 [0.68, 3.82]	0.28
Hoarseness	11/253	4.35%	18/251	7.17%	0.61 [0.29, 1.26]	0.18
Sore throat	11/253	4.35%	8/251	3.19%	1.36 [0.56, 3.33]	0.50
Voice alterations	10/253	3.95%	11/251	4.38%	0.90 [0.39, 2.09]	0.81
Dysgeusia	9/253	3.56%	2/251	0.80%	4.46 [0.97, 20.46]	0.05
Neck pain	9/253	3.56%	7/251	2.79%	1.28 [0.48, 3.37]	0.62
Hypothyroidism	7/253	2.77%	4/251	1.59%	1.74 [0.51, 5.86]	0.37
Tinnitus	7/253	2.77%	6/251	2.39%	1.16 [0.39, 3.40]	0.79
Salivary duct inflammation	3/253	1.19%	0/251	0.00%	6.94 [0.36, 133.76]	0.20

AE, Adverse event; CI, confidence interval; P, Probability; RR, Risk ratio.

### Death analysis

The mortality was similar between the two groups. (RR: 1.28 [0.48, 3.41], P = 0.62). The most common cause of death in both groups was the development of a second new cancer (**Table 3**).

**Table 3:** Death analysis.

Death analysis	Radioiodine		Non-radioiodine		Risk ratio [95% CI]	P
	Event/total	%	Event/total	%		
Total	9/642	1.40%	7/638	1.10%	1.28 [0.48, 3.41]	0.62
New primary cancer	4/642	0.62%	4/638	0.63%	0.99 [0.25, 3.96]	0.99
Myocardial infarction	1/642	0.16%	1/638	0.16%	0.99 [0.06, 15.77]	1.00
Lung disease	1/642	0.16%	0/638	0.00%	2.98 [0.12, 73.04]	0.50
Liver disease	1/642	0.16%	1/638	0.16%	0.99 [0.06, 15.85]	1.00
Heart failure	0/642	0.00%	1/638	0.16%	0.33 [0.01, 8.08]	0.50
Others	2/642	0.31%	0/638	0.00%	4.96 [0.24, 102.81]	0.30

CI, confidence interval; P, Probability; RR, Risk ratio.

### Second new cancers

Total rate of second new cancers was similar between the two groups (RR: 1.26 [0.58, 2.73], P = 0.55). The most common second new cancer in both groups was breast cancer (**Table 4**).

**Table 4:** Second new cancers. .

Second new cancers	Radioiodine		Non-radioiodine		Risk ratio [95% CI]	P
	Event/total	%	Event/total	%		
Total	14/253	5.53%	11/251	4.38%	1.26 [0.58, 2.73]	0.55
Breast	8/253	3.16%	5/251	1.99%	1.59 [0.53, 4.79]	0.41
Basal cell carcinoma	2/253	0.79%	1/251	0.40%	1.98 [0.18, 21.74]	0.57
Head and neck	1/253	0.40%	0/251	0.00%	2.98 [0.12, 72.72]	0.50
Lymphoma	1/253	0.40%	0/251	0.00%	2.98 [0.12, 72.72]	0.50
Multiple myeloma	1/253	0.40%	2/251	0.80%	0.50 [0.05, 5.44]	0.57
Rectal	1/253	0.40%	0/251	0.00%	2.98 [0.12, 72.72]	0.50
Chronic lymphocytic leukaemia	0/253	0.00%	1/251	0.40%	0.33 [0.01, 8.08]	0.50
Neuroendocrine/lung	0/253	0.00%	1/251	0.40%	0.33 [0.01, 8.08]	0.50
Prostate	0/253	0.00%	1/251	0.40%	0.33 [0.01, 8.08]	0.50

CI, confidence interval; P, Probability; RR, Risk ratio.

### Publication bias

**Figure 5:**
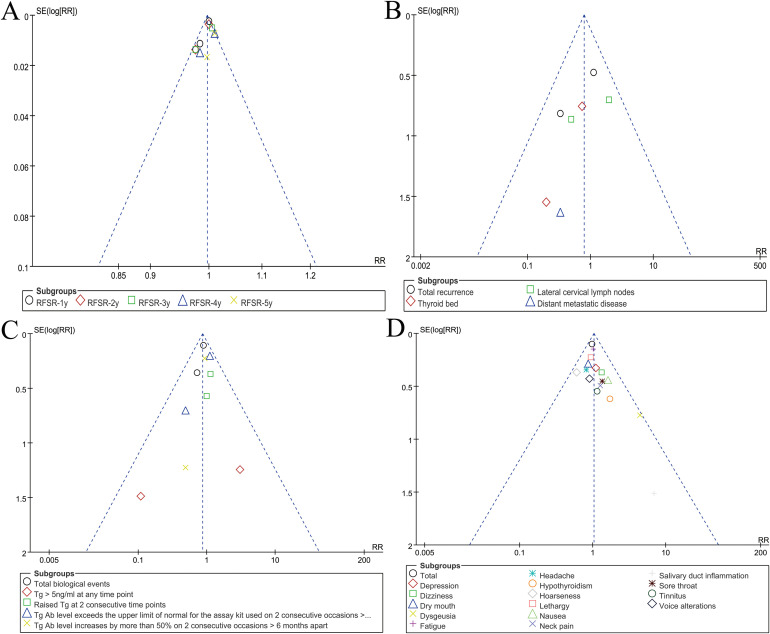
Funnel plots of RFSR **(A)**, recurrence **(B)**, biological events **(C)**, and AEs summary **(D)**.

Visual inspection of funnel plots for RFSR, recurrence, biological outcomes, and adverse events indicated low likelihood of reporting bias (**Figure 5**).

## Discussion

The optimal management strategy for DTC following thyroidectomy has remained a point of clinical controversy for years. While total thyroidectomy or lobectomy alone often provides excellent long-term outcomes for low-risk patients, the historical practice of routinely administering postoperative radioiodine to ablate remnant thyroid tissue has persisted in many clinical settings (18). This persistence is partly due to legacy protocols and partly due to uncertainty regarding the true benefits of radioiodine in preventing disease recurrence. Recent advancements in diagnostic surveillance, risk stratification, and molecular understanding of DTC have further challenged the necessity of radioiodine in low-risk settings. Despite evolving guidelines, such as the 2015 ATA recommendation to limit radioiodine use in low-risk patients, practice patterns remain heterogeneous (10). Thus, a high-quality synthesis of the best available randomized evidence was urgently needed. This meta-analysis, focusing exclusively on phase 3 RCTs—ESTIMABL2 and IoN—addresses this gap (11, 12). Our pooled analysis involving 1,280 patients found no significant benefit of radioiodine in reducing recurrence rates, improving RFS, or altering structural and biological event rates. Furthermore, no differences were found in terms of AEs, mortality, or secondary malignancies, reinforcing the argument against routine radioiodine administration in this patient population.

The question of recurrence lies at the heart of the ongoing debate regarding the necessity of radioiodine in low-risk DTC. In our meta-analysis, we found that radioiodine therapy did not confer a statistically significant reduction in recurrence risk, nor did it improve DFS. These findings not only echo the results of the ESTIMABL2 and IoN trials individually but also align with accumulating observational evidence suggesting that the natural course of low-risk DTC is inherently favorable, regardless of adjuvant radioiodine (11, 12). One important consideration is the absolute recurrence rate in both groups, which was below 5%, suggesting that recurrence in this subgroup is a rare event. The implication is that even if radioiodine were to reduce recurrence marginally, the clinical relevance would remain limited due to the already low baseline risk (19). Moreover, the site of recurrence—whether local, regional, or distant—did not differ significantly between the radioiodine and non- radioiodine arms in our analysis. This observation counters the traditional rationale that radioiodine may reduce microscopic distant metastasis that escapes surgical excision. In the current era of high-resolution ultrasound and sensitive serum Tg assays, the early detection of recurrence is more achievable, potentially reducing the clinical need for radioiodine as a prophylactic tool (20). Some experts have also proposed that radioiodine might selectively benefit subgroups of low-risk patients, such as those with multifocality, microscopic lymphovascular invasion, or younger age, but such hypotheses remain unproven in randomized settings (21). Another important angle is the temporal pattern of recurrence. Our data show no difference in RFS over a 5-year follow-up period, suggesting that recurrences, when they do occur, are not only rare but also unlikely to be temporally delayed by radioiodine. In both the ESTIMABL2 and IoN trials, recurrence was primarily defined using surrogate criteria—such as abnormal neck imaging findings or elevated serum thyroglobulin (Tg) or anti-Tg antibodies—rather than histopathologic confirmation. This reflects a well-recognized challenge in low-risk DTC, where many so-called “recurrences” represent indolent or biochemical findings that may not necessitate therapeutic intervention. Accordingly, recurrence in this context should be interpreted as a composite surrogate endpoint rather than a strictly pathological event, which may partly explain the minimal clinical impact observed in our pooled analysis (11, 12). This undermines the argument for radioiodine as a long-term protective measure. Additionally, modern risk-adapted surveillance protocols—such as dynamic risk stratification based on postoperative Tg trends and imaging—allow for more individualized follow-up and delayed intervention strategies, which further diminishes the value of a blanket radioiodine policy (22). Ultimately, these findings reinforce the view that recurrence should not be the principal justification for administering radioiodine in low-risk DTC.

In addition to recurrence, structural and biological events serve as important surrogate endpoints in monitoring patients after thyroidectomy. In our meta-analysis, structural events—defined as radiologic or cytologic findings suggestive of persistent or recurrent disease—were numerically lower in the radioiodine group, but the difference did not reach statistical significance. Similarly, biological events—characterized by elevated levels of serum Tg or TgAb in the absence of structural disease—were also not significantly different between groups. These findings challenge the notion that radioiodine contributes meaningfully to reducing biochemical or structural disease burden in low-risk patients (23). The slightly higher frequency of Tg > 5 ng/mL in the non-radioiodine group may superficially suggest biochemical benefit from radioiodine, but this must be interpreted cautiously. Elevated Tg levels in non-ablated patients may reflect residual normal thyroid tissue rather than recurrent disease. Importantly, this distinction becomes clinically relevant only if the elevated Tg leads to a change in patient management—such as unnecessary imaging, biopsies, or anxiety—which can be mitigated by physician awareness and patient education (23). Moreover, it is worth emphasizing that Tg kinetics over time (e.g., declining or stable trends) are often more informative than absolute values, especially when interpreted alongside imaging findings (24). Another consideration is the effect of radioiodine on diagnostic clarity. While ablation of remnant thyroid tissue may simplify biochemical monitoring, modern assay sensitivity and imaging capabilities allow for effective surveillance even in non-ablated patients. In fact, updated guidelines from the ATA and European Thyroid Association (ETA) now endorse the omission of radioiodine in low-risk patients, partly on the basis of these technological advances (10, 25). In clinical practice, the decision to administer radioiodine should not be driven solely by the desire for biochemical clarity, especially if it comes at the cost of exposing patients to radiation without improving hard outcomes. Furthermore, the absence of significant differences in structural and biological event rates raises questions about the long-term benefits of radioiodine (26). If radioiodine does not appreciably reduce persistent or recurrent structural disease, nor influence biochemical markers in a clinically actionable way, then its utility in this context becomes increasingly tenuous. This is particularly relevant as healthcare systems strive to balance efficacy with cost and safety, and as patient-centered care models prioritize shared decision-making and quality of life.

The safety profile of radioiodine has historically been a concern, particularly regarding both acute side effects and long-term sequelae. In our analysis, we found no statistically significant difference in the overall incidence of AEs between the radioiodine and non- radioiodine groups, nor in the incidence of grade 3–5 AEs. The most frequently reported AEs in the radioiodine group were fatigue (25.7%), lethargy (12.7%), and dry mouth (8.3%)—all of which are consistent with transient, non-life-threatening symptoms associated with salivary gland irradiation. Dysgeusia also tended to occur more frequently in the radioiodine group (3.6% vs 0.8%), with a relative risk of 4.46 (p = 0.05). This trend is biologically plausible, as radioiodine uptake by the salivary glands and gustatory epithelium can transiently disrupt taste perception through localized radiation-induced inflammation or ductal damage. Importantly, dysgeusia is generally mild and self-limited, resolving within weeks to months after treatment, and does not typically require medical intervention. Nevertheless, this observation underscores the need for patient counseling regarding temporary sensory disturbances following RAI (27). These findings are clinically important, as they support the assertion that radioiodine, when used judiciously, is a well-tolerated therapy in most patients (28). However, the absence of significant differences in AEs should not necessarily be interpreted as justification for routine use. Even mild or moderate symptoms can adversely affect patient quality of life, particularly when they occur in patients who are unlikely to derive measurable benefit from the intervention (29). Additionally, the possibility of rare but serious complications—such as sialadenitis, lacrimal gland dysfunction, or transient infertility—remains a theoretical concern, especially in younger patients or those receiving repeat doses of radioiodine (30). Mortality was also comparable between groups, and no thyroid cancer-related deaths were reported. This reinforces the well-established notion that low-risk DTC has an exceedingly favorable prognosis and that death is a rare event, further calling into question the need for aggressive adjuvant therapy (31). Notably, the most common cause of death in both arms was second primary malignancy, which leads to the next point of discussion. The incidence of second primary cancers, a key long-term safety concern with radioactive exposure, was not significantly increased in the radioiodine group. Previous retrospective studies have raised alarm over possible associations between radioiodine and subsequent hematologic or solid tumors (32). However, our findings, derived from prospective RCTs, are more reassuring. The most commonly observed second cancer was breast cancer—likely reflecting baseline population prevalence rather than treatment-induced risk. It is plausible that modern radioiodine dosing strategies, which typically involve lower activities and risk-adapted indications, mitigate the potential for carcinogenicity (33). Nevertheless, given the latency of radiation-induced malignancies, ongoing long-term surveillance in this population remains essential. In conclusion, our analysis indicates that radioiodine does not meaningfully increase the risk of acute or chronic toxicity, death, or second malignancy in low-risk DTC. While this supports the safety of radioiodine in appropriately selected patients, it also underscores the lack of compelling justification for its routine use in a population unlikely to benefit. Clinicians should weigh these findings against patient-specific factors and preferences when discussing adjuvant treatment options.

Despite the strengths of this meta-analysis, several limitations warrant consideration. First, the number of included RCTs remains limited to two, although they represent the highest quality evidence currently available. Second, the follow-up durations in both trials, while adequate to detect early recurrence, may be insufficient to capture very late recurrences or long-term sequelae such as chronic toxicity or secondary cancers. Third, subgroup analyses based on tumor histology, patient age, gender, or surgical extent were not possible due to lack of granular data, which may limit the generalizability of the findings across diverse patient populations. Additionally, patient adherence to follow-up protocols, variability in Tg assay sensitivity, and institutional differences in imaging thresholds could all introduce confounding biases. Another limitation relates to the definition of recurrence in the included RCTs, which was largely based on imaging or biochemical criteria rather than surgical or pathological confirmation. As a result, some recurrences may reflect indolent biochemical findings without clinical relevance, potentially leading to an overestimation of event rates. Lastly, while statistical heterogeneity was low, clinical heterogeneity cannot be entirely excluded given differing national practices, healthcare settings, and patient expectations across the French and UK cohorts.

## Conclusion

The radioiodine therapy does not significantly reduce recurrence or improve RFS in patients with low-risk DTC following thyroidectomy. Structural and biological event rates were comparable between groups, suggesting that modern surveillance methods are effective even without radioiodine. There were no meaningful differences in AEs, mortality, or secondary malignancies, reinforcing the safety of omitting radioiodine in appropriately selected patients. These findings support a risk-adapted, individualized approach and advocate for de-escalation of radioiodine use in the management of low-risk DTC.

## Data Availability

The original contributions presented in the study are included in the article/**Supplementary Material**. Further inquiries can be directed to the corresponding author.

## Supplementary material

Supplementary Figure 1 Cochrane Risk Assessment.

Supplementary Figure 2 Forest plots of RFSR at 1–5 years associated with radioiodine versus non-radioiodine.

Supplementary Figure 3 Comparisons of RFSR associated with radioiodine versus non-radioiodine. **(A)** RFSR at 1–5 years; **(B)** Trend of risk ratios in RFSR.

Supplementary Figure 4 Forest plots of biological events associated with radioiodine versus non-radioiodine.

Supplementary Figure 5 Forest plots of structural events associated with radioiodine versus non-radioiodine.
