# Supplementary figures and images for: Is radioiodine necessary for patients with low-risk differentiated thyroid cancer after thyroidectomy: a pooled analysis of ESTIMABL2 and IoN trials
Source: Front Oncol. 2025 Oct 28;15:1670978. doi: 10.3389/fonc.2025.1670978 (PMC12602227; doi:10.3389/fonc.2025.1670978)

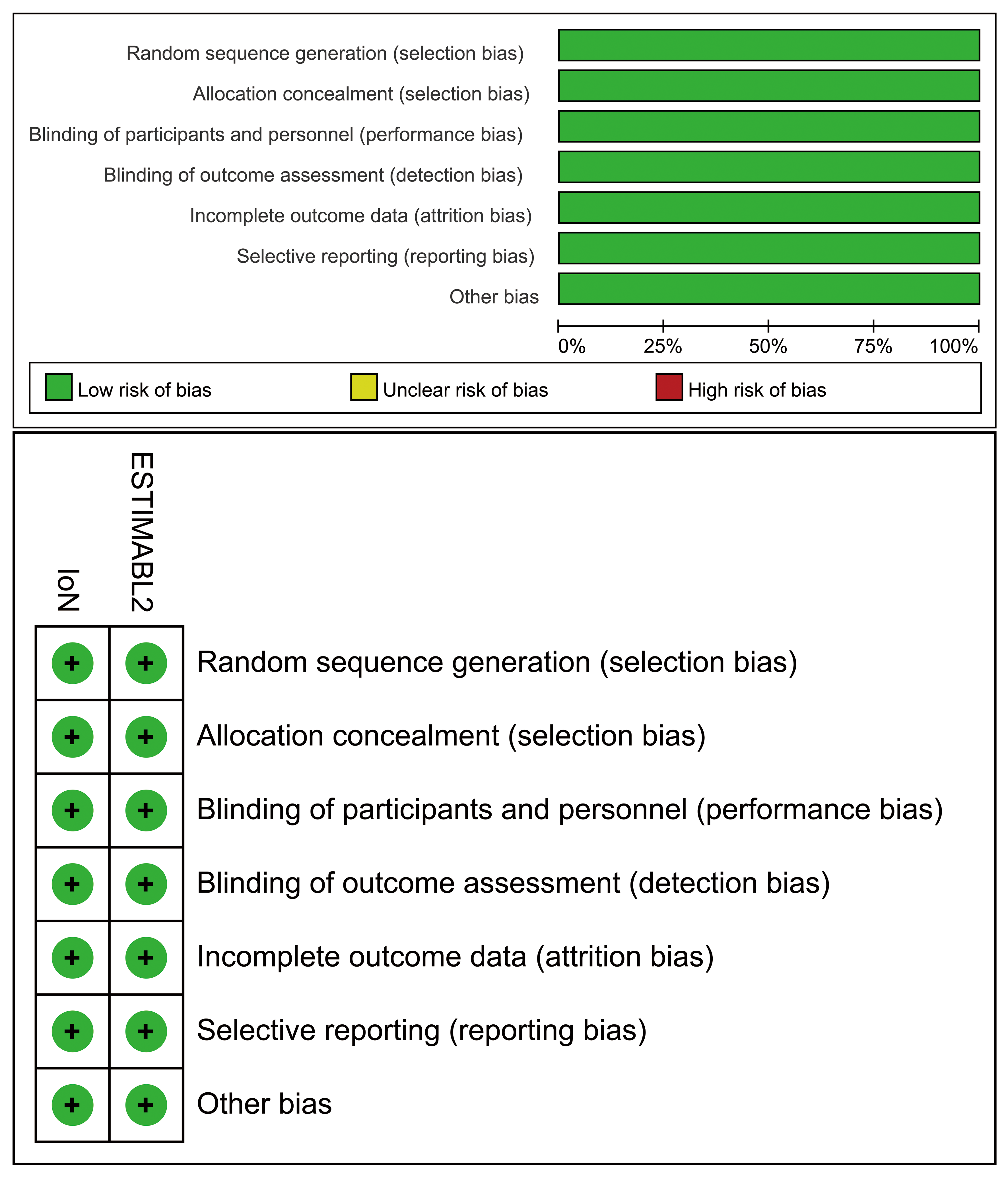

Supplement: Supplementary Figure 1 — Cochrane Risk Assessment. [file Image1.tif]

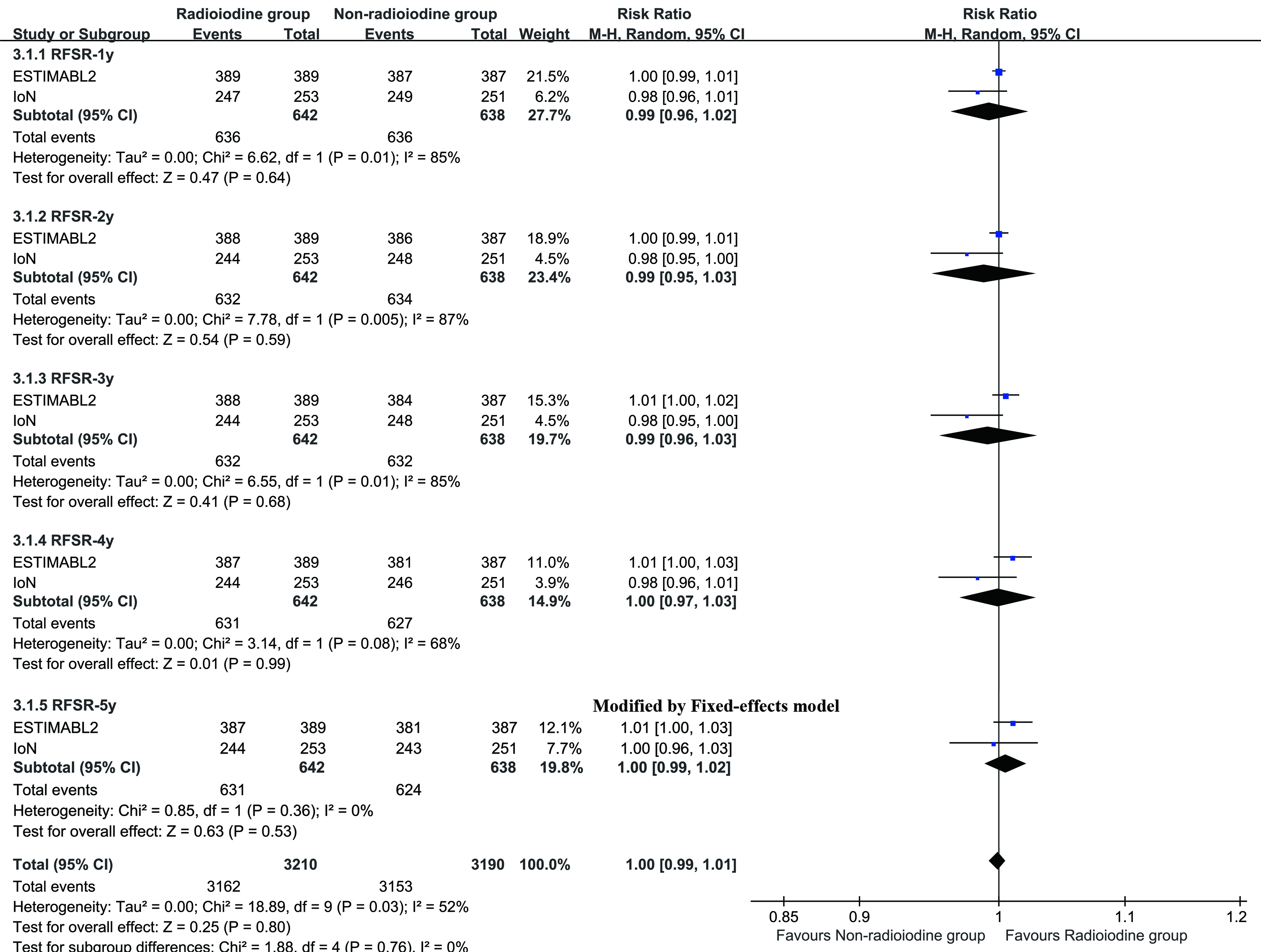

Supplement: Supplementary Figure 2 — Forest plots of RFSR at 1–5 years associated with radioiodine versus non-radioiodine. [file Image2.tif]

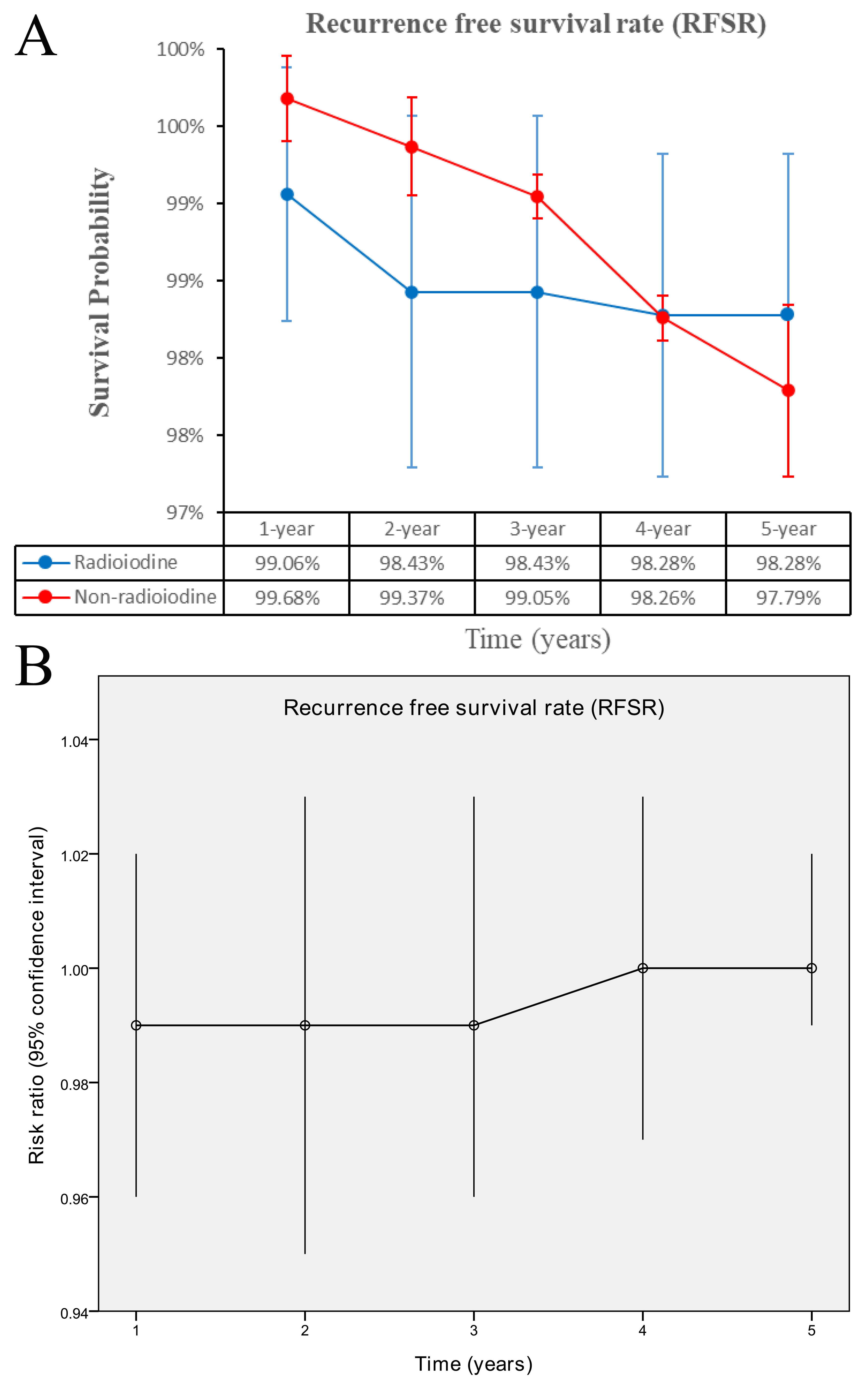

Supplement: Supplementary Figure 3 — Comparisons of RFSR associated with radioiodine versus non-radioiodine. (A) RFSR at 1–5 years; (B) Trend of risk ratios in RFSR. [file Image3.tif]

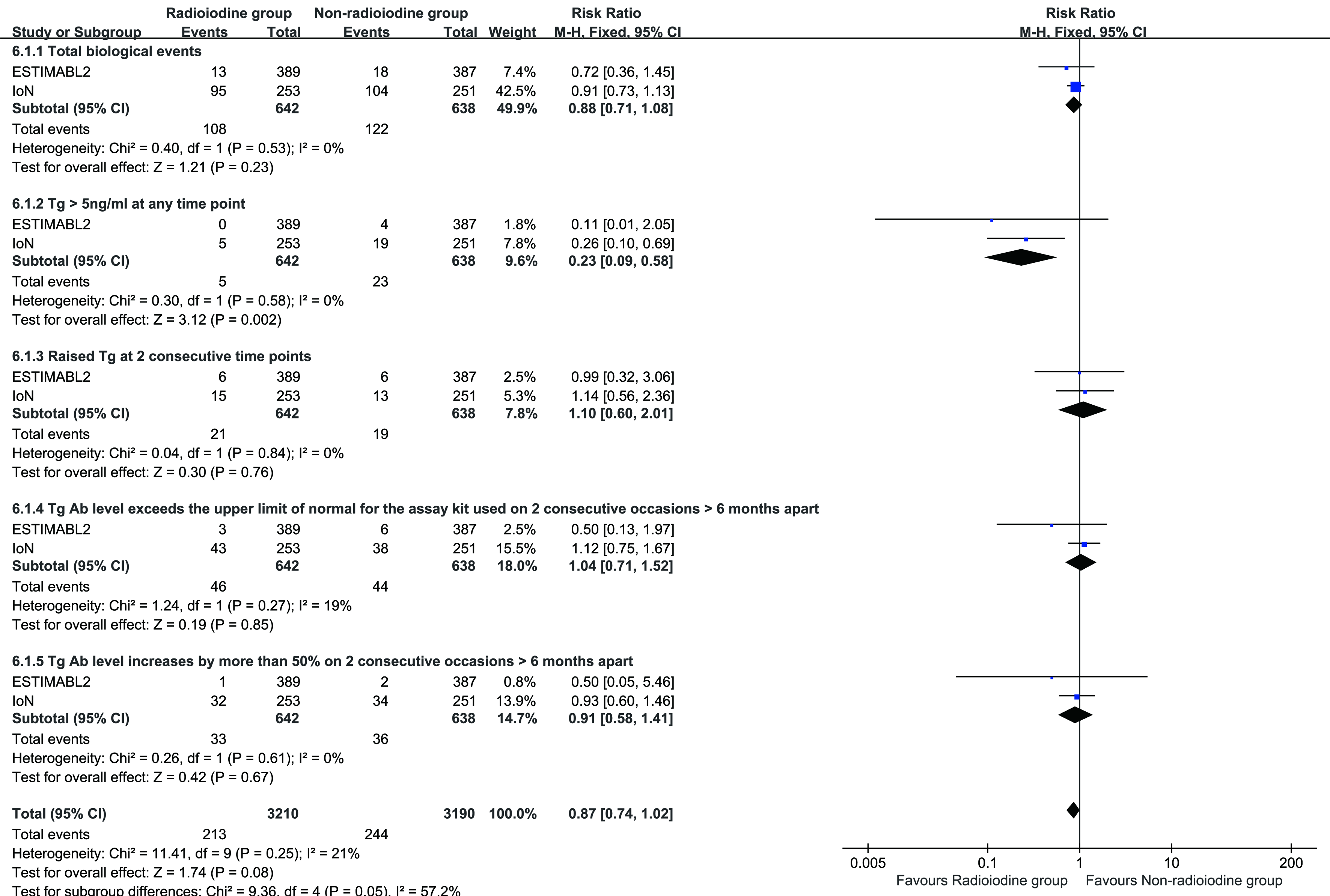

Supplement: Supplementary Figure 4 — Forest plots of biological events associated with radioiodine versus non-radioiodine. [file Image4.tif]

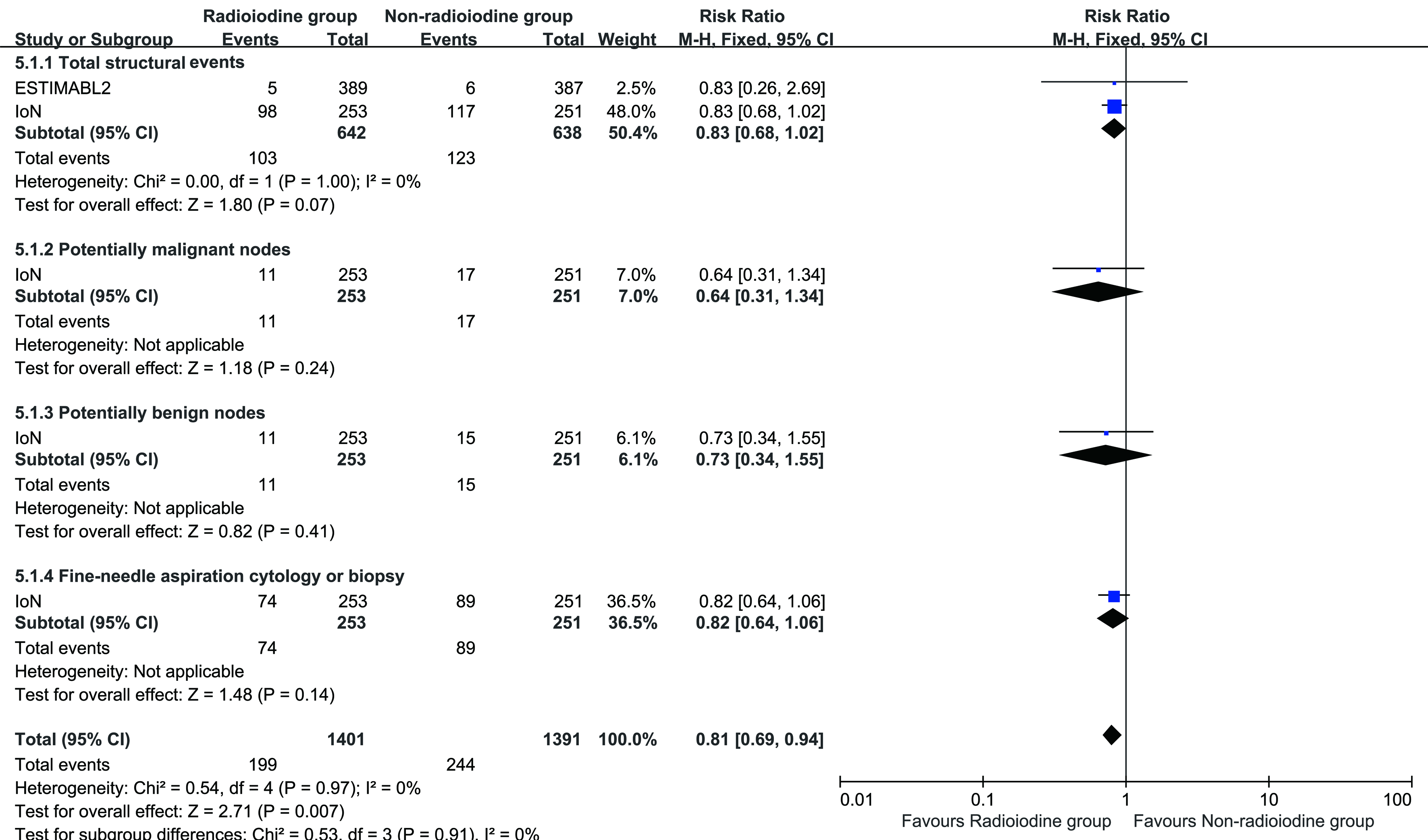

Supplement: Supplementary Figure 5 — Forest plots of structural events associated with radioiodine versus non-radioiodine. [file Image5.tif]
