# Supplementary material for: Is radioiodine necessary for patients with low-risk differentiated thyroid cancer after thyroidectomy: a pooled analysis of ESTIMABL2 and IoN trials
Source: Front Oncol. 2025 Oct 28;15:1670978. doi: 10.3389/fonc.2025.1670978 (PMC12602227; doi:10.3389/fonc.2025.1670978)
Supplement: Supplementary file 6 [file Table1.docx]

**Table S1** Search strategy.

| **PubMed**  The database was searched on June 20, 2025, n=347.  Search Strategy:  **#1 Search: Radioiodine[Title/Abstract] OR Radioactive Iodine Therapy[Title/Abstract] OR Iodine[Title/Abstract] Sort by: Most Recent n = 74800**  **#2 Search: Neoplasm, Thyroid[Title/Abstract] OR Thyroid Neoplasm[Title/Abstract] OR Neoplasms, Thyroid[Title/Abstract] OR Thyroid Carcinoma[Title/Abstract] OR Carcinomas, Thyroid[Title/Abstract] OR Carcinoma, Thyroid[Title/Abstract] OR Thyroid Carcinomas[Title/Abstract] OR Cancer of the Thyroid[Title/Abstract] OR Cancer of Thyroid[Title/Abstract] OR Thyroid Cancers[Title/Abstract] OR Thyroid Cancer[Title/Abstract] OR Cancers, Thyroid[Title/Abstract] OR Cancer, Thyroid[Title/Abstract] OR Thyroid Adenoma[Title/Abstract] OR Adenomas, Thyroid[Title/Abstract] OR Adenoma, Thyroid[Title/Abstract] OR Thyroid Adenomas[Title/Abstract] Sort by: Most Recent n = 57967**  **#3 Search: Randomized[Title/Abstract] OR Randomly[Title/Abstract] OR Randomised[Title/Abstract] Sort by: Most Recent n = 1260968**  **#1 and #2 and #3 n = 347** |
| --- |
| **Web of Science**  The database was searched on June 20, 2025, n=318.  Search Strategy:  Radioiodine OR Radioactive Iodine Therapy OR Iodine (Abstract) AND Neoplasm, Thyroid OR Thyroid Neoplasm OR Neoplasms, Thyroid OR Thyroid Carcinoma OR Carcinomas, Thyroid OR Carcinoma, Thyroid OR Thyroid Carcinomas OR Cancer of the Thyroid OR Cancer of Thyroid OR Thyroid Cancers OR Thyroid Cancer OR Cancers, Thyroid OR Cancer, Thyroid OR Thyroid Adenoma OR Adenomas, Thyroid OR Adenoma, Thyroid OR Thyroid Adenomas (Abstract) AND Randomized OR Randomly OR Randomised (Abstract) and Preprint Citation Index (Exclude – Database) |
| **EMBASE**  The database was searched on June 20, 2025, n=683.  Search Strategy:  (Radioiodine:ti,ab,kw OR Radioactive Iodine Therapy:ti,ab,kw OR Iodine:ti,ab,kw) AND (Neoplasm, Thyroid:ti,ab,kw OR Thyroid Neoplasm:ti,ab,kw OR Neoplasms, Thyroid:ti,ab,kw OR Thyroid Carcinoma:ti,ab,kw OR Carcinomas, Thyroid:ti,ab,kw OR Carcinoma, Thyroid:ti,ab,kw OR Thyroid Carcinomas:ti,ab,kw OR Cancer of the Thyroid:ti,ab,kw OR Cancer of Thyroid:ti,ab,kw OR Thyroid Cancers:ti,ab,kw OR Thyroid Cancer:ti,ab,kw OR Cancers, Thyroid:ti,ab,kw OR Cancer, Thyroid:ti,ab,kw OR Thyroid Adenoma:ti,ab,kw OR Adenomas, Thyroid:ti,ab,kw OR Adenoma, Thyroid:ti,ab,kw OR Thyroid Adenomas:ti,ab,kw) AND **(Randomly**:ti,ab,kw **OR Randomised**:ti,ab,kw **OR Randomized** :ti,ab,kw**)** |
| **Cochrane Library**  The database was searched on June 20, 2025, n=35.  Search Strategy:  (Radioiodine OR Radioactive Iodine Therapy OR Iodine**)** in Title Abstract Keyword AND (Neoplasm, Thyroid OR Thyroid Neoplasm OR Neoplasms, Thyroid OR Thyroid Carcinoma OR Carcinomas, Thyroid OR Carcinoma, Thyroid OR Thyroid Carcinomas OR Cancer of the Thyroid OR Cancer of Thyroid OR Thyroid Cancers OR Thyroid Cancer OR Cancers, Thyroid OR Cancer, Thyroid OR Thyroid Adenoma OR Adenomas, Thyroid OR Adenoma, Thyroid OR Thyroid Adenomas**)** in Title Abstract Keyword AND (**Randomized OR Randomly OR Randomised)** in Title Abstract Keyword - (Word variations have been searched) |
| **ScienceDirect**  The database was searched on June 20, 2025, n=822.  Search Strategy:  Title, abstract, keywords: ((“Radioiodine” OR “Radioactive Iodine Therapy” OR “Iodine”) AND (“Neoplasm, Thyroid” OR “Thyroid Neoplasm” OR “Neoplasms, Thyroid” OR “Thyroid Carcinoma” OR “Carcinomas, Thyroid” OR “Carcinoma, Thyroid” OR “Thyroid Carcinomas” OR “Cancer of the Thyroid” OR “Cancer of Thyroid” OR “Thyroid Cancers” OR “Thyroid Cancer” OR “Cancers, Thyroid” OR “Cancer, Thyroid” OR “Thyroid Adenoma” OR “Adenomas, Thyroid” OR “Adenoma, Thyroid” OR “Thyroid Adenomas”) AND (“**Randomized**” **OR Randomly**” **OR** “**Randomised**”)) |
| **Scopus**  The database was searched on June 20, 2025, n=288.  Search Strategy:  (TITLE-ABS-KEY (Radioiodine OR Radioactive Iodine Therapy OR Iodine) AND TITLE-ABS-KEY (Neoplasm, Thyroid OR Thyroid Neoplasm OR Neoplasms, Thyroid OR Thyroid Carcinoma OR Carcinomas, Thyroid OR Carcinoma, Thyroid OR Thyroid Carcinomas OR Cancer of the Thyroid OR Cancer of Thyroid OR Thyroid Cancers OR Thyroid Cancer OR Cancers, Thyroid OR Cancer, Thyroid OR Thyroid Adenoma OR Adenomas, Thyroid OR Adenoma, Thyroid OR Thyroid Adenomas) AND TITLE-ABS-KEY (Randomized OR Randomly OR Randomised)) |

**Note:** The combined text and medical subject heading (MeSH) terms used were: “**Radioiodine**”, “Thyroid Cancer”, and “**Randomized**”.
