# Supplementary material for: Is radioiodine necessary for patients with low-risk differentiated thyroid cancer after thyroidectomy: a pooled analysis of ESTIMABL2 and IoN trials
Source: Front Oncol. 2025 Oct 28;15:1670978. doi: 10.3389/fonc.2025.1670978 (PMC12602227; doi:10.3389/fonc.2025.1670978)
Supplement: Supplementary file 7 [file Table2.doc]

**Table S2 Methodological quality assessments (Jadad scale) of the included studies.**

| **Study** | **Randomization** | **Concealment of allocation** | **Double blinding** | **Withdrawals and dropouts** | **Quality (score)** |
| --- | --- | --- | --- | --- | --- |
| ESTIMABL2 [11,17] | ** | ** | * | * | 7 |
| IoN [12] | ** | ** | * | * | 7 |
