# Supplementary material for: Is radioiodine necessary for patients with low-risk differentiated thyroid cancer after thyroidectomy: a pooled analysis of ESTIMABL2 and IoN trials
Source: Front Oncol. 2025 Oct 28;15:1670978. doi: 10.3389/fonc.2025.1670978 (PMC12602227; doi:10.3389/fonc.2025.1670978)
Supplement: Supplementary file 8 [file Table3.doc]

**Table S3** GRADE quality assessment by therapeutic strategy and study design for the outcomes.

| **Outcomes** | **No. of Participants** | | **Differences (95%CI) ^a^** | **Quality Assessment** | | | | | **Quality** |
| --- | --- | --- | --- | --- | --- | --- | --- | --- | --- |
|  | **Radioiodine** | **Non-radioiodine** |  | **Risk of Bias^b^** | **Inconsistency** | **Indirectness** | **Imprecision** | **Publication Bias^c^** |  |
| **Survival** |  |  |  |  |  |  |  |  |  |
| RFS | 1280 | | 0.96 [0.80, 1.15] | Low | No inconsistency | No indirectness | No imprecision | Unlikely | High |
| **Survival rate** |  |  |  |  |  |  |  |  |  |
| **RFSR** |  |  |  |  |  |  |  |  |  |
| RFSR-1y | 636/642 | 636/638 | 0.99 [0.96, 1.02] | Low | Serious (-1) | No indirectness | No imprecision | Unlikely | Medium |
| RFSR-2y | 632/642 | 634/638 | 0.99 [0.95, 1.03] | Low | Serious (-2) | No indirectness | No imprecision | Unlikely | Medium |
| RFSR-3y | 632/642 | 632/638 | 0.99 [0.96, 1.03] | Low | Serious (-3) | No indirectness | No imprecision | Unlikely | Medium |
| RFSR-4y | 631/642 | 627/638 | 1.00 [0.97, 1.03] | Low | Serious (-4) | No indirectness | No imprecision | Unlikely | Medium |
| RFSR-5y | 631/642 | 624/638 | 1.00 [0.99, 1.02] | Low | No inconsistency | No indirectness | No imprecision | Unlikely | High |
| **Recurrence** |  | |  |  |  |  |  |  |  |
| Total | 11/642 | 14/638 | 0.78 [0.36, 1.70] | Low | No inconsistency | No indirectness | No imprecision | Unlikely | High |
| Thyroid bed | 3/642 | 6/638 | 0.53 [0.15, 1.95] | Low | No inconsistency | No indirectness | No imprecision | Unlikely | High |
| Lateral cervical lymph nodes | 8/642 | 7/638 | 1.14 [0.41, 3.11] | Low | No inconsistency | No indirectness | No imprecision | Unlikely | High |
| Distant metastatic disease | 0/642 | 1/638 | 0.33 [0.01, 8.08] | Low | No inconsistency | No indirectness | No imprecision | Unlikely | High |
| **Structural events** |  | |  |  |  |  |  |  |  |
| Total structural events | 103/642 | 123/638 | 0.83 [0.68, 1.02] | Low | No inconsistency | No indirectness | No imprecision | Unlikely | High |
| Potentially malignant nodes | 11/253 | 17/251 | 0.64 [0.31, 1.34] | Low | No inconsistency | No indirectness | No imprecision | Unlikely | High |
| Potentially benign nodes | 11/253 | 15/251 | 0.73 [0.34, 1.55] | Low | No inconsistency | No indirectness | No imprecision | Unlikely | High |
| Fine-needle aspiration cytology or biopsy | 74/253 | 89/251 | 0.82 [0.64, 1.06] | Low | No inconsistency | No indirectness | No imprecision | Unlikely | High |
| **Biological events** |  | |  |  |  |  |  |  |  |
| Total biological events | 108/642 | 122/638 | 0.88 [0.71, 1.08] | Low | No inconsistency | No indirectness | No imprecision | Unlikely | High |
| Tg > 5ng/ml at any time point | 5/642 | 23/638 | 0.23 [0.09, 0.58] | Low | No inconsistency | No indirectness | No imprecision | Unlikely | High |
| Raised Tg at 2 consecutive time points | 21/642 | 19/638 | 1.10 [0.60, 2.01] | Low | No inconsistency | No indirectness | No imprecision | Unlikely | High |
| TgAb level exceeds the upper limit of normal for the assay kit used on 2 consecutive occasions > 6 months apart | 46/642 | 44/638 | 1.04 [0.71, 1.52] | Low | No inconsistency | No indirectness | No imprecision | Unlikely | High |
| TgAb level increases by more than 50% on 2 consecutive occasions > 6 months apart | 33/642 | 36/638 | 0.91 [0.58, 1.41] | Low | No inconsistency | No indirectness | No imprecision | Unlikely | High |
| **Any grade adverse events** |  | |  |  |  |  |  |  |  |
| Total | 103/253 | 105/251 | 0.97 [0.79, 1.20] | Low | No inconsistency | No indirectness | No imprecision | Unlikely | High |
| Fatigue | 65/253 | 63/251 | 1.02 [0.76, 1.38] | Low | No inconsistency | No indirectness | No imprecision | Unlikely | High |
| Lethargy | 32/253 | 34/251 | 0.93 [0.60, 1.46] | Low | No inconsistency | No indirectness | No imprecision | Unlikely | High |
| Dry mouth | 21/253 | 24/251 | 0.87 [0.50, 1.52] | Low | No inconsistency | No indirectness | No imprecision | Unlikely | High |
| Depression | 18/253 | 16/251 | 1.12 [0.58, 2.14] | Low | No inconsistency | No indirectness | No imprecision | Unlikely | High |
| Dizziness | 16/253 | 12/251 | 1.32 [0.64, 2.74] | Low | No inconsistency | No indirectness | No imprecision | Unlikely | High |
| Headache | 14/253 | 17/251 | 0.82 [0.41, 1.62] | Low | No inconsistency | No indirectness | No imprecision | Unlikely | High |
| Nausea | 13/253 | 8/251 | 1.61 [0.68, 3.82] | Low | No inconsistency | No indirectness | No imprecision | Unlikely | High |
| Hoarseness | 11/253 | 18/251 | 0.61 [0.29, 1.26] | Low | No inconsistency | No indirectness | No imprecision | Unlikely | High |
| Sore throat | 11/253 | 8/251 | 1.36 [0.56, 3.33] | Low | No inconsistency | No indirectness | No imprecision | Unlikely | High |
| Voice alterations | 10/253 | 11/251 | 0.90 [0.39, 2.09] | Low | No inconsistency | No indirectness | No imprecision | Unlikely | High |
| Dysgeusia | 9/253 | 2/251 | 4.46 [0.97, 20.46] | Low | No inconsistency | No indirectness | No imprecision | Unlikely | High |
| Neck pain | 9/253 | 7/251 | 1.28 [0.48, 3.37] | Low | No inconsistency | No indirectness | No imprecision | Unlikely | High |
| Hypothyroidism | 7/253 | 4/251 | 1.74 [0.51, 5.86] | Low | No inconsistency | No indirectness | No imprecision | Unlikely | High |
| Tinnitus | 7/253 | 6/251 | 1.16 [0.39, 3.40] | Low | No inconsistency | No indirectness | No imprecision | Unlikely | High |
| Salivary duct inflammation | 3/253 | 0/251 | 6.94 [0.36, 133.76] | Low | No inconsistency | No indirectness | No imprecision | Unlikely | High |
| **Grade 3-5 adverse events** |  | |  |  |  |  |  |  |  |
| Total | 1/253 | 4/251 | 0.25 [0.03, 2.20] | Low | No inconsistency | No indirectness | No imprecision | Unlikely | High |
| Lethargy | 1/253 | 2/251 | 0.50 [0.05, 5.44] | Low | No inconsistency | No indirectness | No imprecision | Unlikely | High |
| Dizziness | 0/253 | 1/251 | 0.33 [0.01, 8.08] | Low | No inconsistency | No indirectness | No imprecision | Unlikely | High |
| Fatigue | 0/253 | 2/251 | 0.20 [0.01, 4.11] | Low | No inconsistency | No indirectness | No imprecision | Unlikely | High |
| Tinnitus | 0/253 | 1/251 | 0.33 [0.01, 8.08] | Low | No inconsistency | No indirectness | No imprecision | Unlikely | High |
| **Death analysis** |  | |  |  |  |  |  |  |  |
| Total | 9/642 | 7/638 | 1.28 [0.48, 3.41] | Low | No inconsistency | No indirectness | No imprecision | Unlikely | High |
| Second new cancer | 4/642 | 4/638 | 0.99 [0.25, 3.96] | Low | No inconsistency | No indirectness | No imprecision | Unlikely | High |
| Myocardial infarction | 1/642 | 1/638 | 0.99 [0.06, 15.77] | Low | No inconsistency | No indirectness | No imprecision | Unlikely | High |
| Lung disease | 1/642 | 0/638 | 2.98 [0.12, 73.04] | Low | No inconsistency | No indirectness | No imprecision | Unlikely | High |
| Liver disease | 1/642 | 1/638 | 0.99 [0.06, 15.85] | Low | No inconsistency | No indirectness | No imprecision | Unlikely | High |
| Heart failure | 0/642 | 1/638 | 0.33 [0.01, 8.08] | Low | No inconsistency | No indirectness | No imprecision | Unlikely | High |
| Others | 2/642 | 0/638 | 4.96 [0.24, 102.81] | Low | No inconsistency | No indirectness | No imprecision | Unlikely | High |
| **Second new cancers** |  | |  |  |  |  |  |  |  |
| Total | 14/253 | 11/251 | 1.26 [0.58, 2.73] | Low | No inconsistency | No indirectness | No imprecision | Unlikely | High |
| Breast | 8/253 | 5/251 | 1.59 [0.53, 4.79] | Low | No inconsistency | No indirectness | No imprecision | Unlikely | High |
| Basal cell carcinoma | 2/253 | 1/251 | 1.98 [0.18, 21.74] | Low | No inconsistency | No indirectness | No imprecision | Unlikely | High |
| Head and neck | 1/253 | 0/251 | 2.98 [0.12, 72.72] | Low | No inconsistency | No indirectness | No imprecision | Unlikely | High |
| Lymphoma | 1/253 | 0/251 | 2.98 [0.12, 72.72] | Low | No inconsistency | No indirectness | No imprecision | Unlikely | High |
| Multiple myeloma | 1/253 | 2/251 | 0.50 [0.05, 5.44] | Low | No inconsistency | No indirectness | No imprecision | Unlikely | High |
| Rectal | 1/253 | 0/251 | 2.98 [0.12, 72.72] | Low | No inconsistency | No indirectness | No imprecision | Unlikely | High |
| Chronic lymphocytic leukaemia | 0/253 | 1/251 | 0.33 [0.01, 8.08] | Low | No inconsistency | No indirectness | No imprecision | Unlikely | High |
| Neuroendocrine/lung | 0/253 | 1/251 | 0.33 [0.01, 8.08] | Low | No inconsistency | No indirectness | No imprecision | Unlikely | High |
| Prostate | 0/253 | 1/251 | 0.33 [0.01, 8.08] | Low | No inconsistency | No indirectness | No imprecision | Unlikely | High |

**Abbreviations:** AE: Adverse event; CI: Confidence interval; DTC: Differentiated thyroid cancer; GRADE: Grading of Recommendations Assessment, Development and Evaluation; HR: Hazard ratio; P: Probability; RCT: Randomized controlled trial; RFS: Recurrence-free survival; RFSR: Recurrence-free survival rate; RR: Risk ratio; Tg: Thyroglobulin; TgAb: Thyroglobulin antibodies.

^a^ Differences: HR for RFS; RR for RFSR, recurrence, structural events, biological events, death analysis, second new cancers, and AEs.

^b^ Risk of bias assessed using the Jadad scale for randomized controlled trials.

^c^ Publication bias was explored through visual inspection of the funnel plots.
