# Supplementary material for: Is radioiodine necessary for patients with low-risk differentiated thyroid cancer after thyroidectomy: a pooled analysis of ESTIMABL2 and IoN trials
Source: Front Oncol. 2025 Oct 28;15:1670978. doi: 10.3389/fonc.2025.1670978 (PMC12602227; doi:10.3389/fonc.2025.1670978)
Supplement: Supplementary file 9 [file Table4.doc]

**Table S4** Grade 3-5 adverse events.

| **Adverse events** | **Radioiodine** | |  | **Non-radioiodine** | | **Risk ratio [95% CI]** | **P** |
| --- | --- | --- | --- | --- | --- | --- | --- |
|  | **Event/total** | **%** |  | **Event/total** | **%** |  |  |
| **Total** | 1/253 | 0.40% |  | 4/251 | 1.59% | 0.25 [0.03, 2.20] | 0.21 |
| Lethargy | 1/253 | 0.40% |  | 2/251 | 0.80% | 0.50 [0.05, 5.44] | 0.57 |
| Dizziness | 0/253 | 0.00% |  | 1/251 | 0.40% | 0.33 [0.01, 8.08] | 0.50 |
| Fatigue | 0/253 | 0.00% |  | 2/251 | 0.80% | 0.20 [0.01, 4.11] | 0.30 |
| Tinnitus | 0/253 | 0.00% |  | 1/251 | 0.40% | 0.33 [0.01, 8.08] | 0.50 |

**Abbreviations:** AE: Adverse event; CI: confidence interval; P: Probability; RR: Risk ratio.
